# Differential network analysis of bovine muscle reveals changes in gene coexpression patterns in response to changes in maternal nutrition

**DOI:** 10.1186/s12864-020-07068-x

**Published:** 2020-10-02

**Authors:** Lihe Liu, Rocío Amorín, Philipe Moriel, Nicolás DiLorenzo, Phillip A. Lancaster, Francisco Peñagaricano

**Affiliations:** 1 Department of Animal and Dairy Sciences, University of Wisconsin-Madison, 1675 Observatory Drive, Madison, WI 53706 USA; 2 Department of Animal Sciences, University of Florida, Gainesville, FL 32611 USA; 3 Range Cattle Research and Education Center, University of Florida, Ona, FL 33865 USA; 4 North Florida Research and Education Center, University of Florida, Marianna, FL 32351 USA; 5 Department of Clinical Sciences, Kansas State University, Manhattan, KS 66506 USA

**Keywords:** Correlation network, Fetal programming, Maternal diet, Muscle transcriptome

## Abstract

**Background:**

Coexpression network analysis is a powerful tool to reveal transcriptional regulatory mechanisms, identify transcription factors, and discover gene functions. It can also be used to investigate changes in coexpression patterns in response to environmental insults or changes in experimental conditions. Maternal nutrition is considered a major intrauterine regulator of fetal developmental programming. The objective of this study was to investigate structural changes in gene coexpression networks in the muscle of bull beef calves gestated under diets with or without methionine supplementation. Both muscle transcriptome and methylome were evaluated using next generation sequencing.

**Results:**

Maternal methionine supplementation significantly perturbed coexpression patterns in the offspring’s muscle. Indeed, we found that neither the connection strength nor the connectivity pattern of six modules (subnetworks) detected in the control diet were preserved in the methionine-rich diet. Functional characterization revealed that some of the unpreserved modules are implicated in myogenesis, adipogenesis, fibrogenesis, canonical Wnt/β-catenin pathway, ribosome structure, rRNA binding and processing, mitochondrial activities, ATP synthesis and NAD(P) H oxidoreductases, among other functions. The bisulfite sequencing analysis showed that nearly 2% of all evaluated cytosines were differentially methylated between maternal diets. Interestingly, there were significant differences in the levels of gene body DNA methylation between preserved and unpreserved modules.

**Conclusions:**

Overall, our findings provide evidence that maternal nutrition can significantly alter gene coexpression patterns in the offspring, and some of these perturbations are mediated by changes in DNA methylation.

## Background

Transcriptome analysis is an essential tool to uncover the molecular basis of phenotypic variation. The advent of RNA sequencing has dramatically improved the characterization and quantification of transcriptomes [1]. The most common use of RNA sequencing is the identification of differentially expressed genes, that is, genes that show differences in expression between conditions. However, genes and gene products do not usually work in isolation, but they are connected in complex networks. There is increasing interest in moving beyond differential expression and examine transcriptional profiles in the context of molecular networks [2]. It is well-accepted that genes that are controlled by the same set of transcription factors or are involved in the same biological processes tend to have similar expression profiles [3]. This principle is known as guilt-by-association and represents the basis for the reconstruction of gene networks using RNA sequencing data. These networks, commonly called gene coexpression networks, are undirected graphs where nodes correspond to genes and edges represent pairwise expression similarities.

Gene coexpression networks can be used for different purposes. One popular application consists in the characterization of the topology of the reconstructed network and examination of interesting nodes and coexpression structures. This single network analysis focuses on the mechanisms allowing the identification of transcription factors (hub genes), the functional annotation of unknown genes, i.e., the association of genes of unknown function with well-described biological processes, and the detection of transcriptional regulatory programs [4]. Another application consists of evaluating gene coexpression networks but across conditions. Here, the term condition is very broad and can refer to different tissues, different developmental stages, or even different treatments. This application, commonly known as differential network analysis, focuses on determining changes in the topology of the networks across conditions. For instance, it is possible to examine whether connections or subnetworks defined under normal conditions (control group) are reproducible and preserved in the testing group (treatment group) [5]. In this scenario, differences in the topology of these two networks would indicate that coexpression patterns were significantly perturbed by the treatment. Note that expression similarities (coexpression) hint common regulatory mechanisms (coregulation), and hence, changes in the network might indicate that the treatment has disrupted coregulation mechanisms, functional links and biological processes. Undoubtedly, this shift in focus from differentially expressed genes to differentially connected genes provides more holistic insights about gene regulation.

It is well-documented that different intrauterine insults can induce permanent changes to the structure, physiology, and metabolism of the offspring. This phenomenon has been termed fetal programming and may have lasting or lifelong consequences [6]. Maternal nutrition is considered a major intrauterine environmental factor and it is now known that maternal nutritional status during pregnancy can induce remarkable effects on fetal development [7]. There is growing evidence that maternal nutrition can alter epigenetic marks of the fetal genome, such as DNA methylation [8]. Indeed, this link between maternal nutrition and subsequent modification of fetal epigenome, including changes in gene expression, is one of the molecular mechanisms proposed to explain the phenomenon of fetal programming [9].

The main objective of this study was to assess whether maternal nutrition in beef cattle can disrupt gene coexpression patterns in the offspring. Maternal nutritional treatments consisted of control or methionine-rich diets offered during periconceptional and early gestation periods. Both muscle transcriptome and methylome of bull beef calves were evaluated using next generation sequencing. Note that DNA methylation depends on the availability of methyl donors, such as methionine, and hence, we hypothesized that maternal methionine supplementation could alter the fetal epigenome, which in turn could induce significant changes in the topology of gene networks.

## Results

### RNA-sequencing analysis

The RNA-sequencing of the muscle transcriptome yielded about 50 million paired-end reads per sample. Roughly 87% of the reads were mapped to the ARS-UCD1.2 bovine genome assembly using the software Hisat2 (see Additional File 1). After removing highly abundant genes (such as myosins, tropomyosins, and troponins) and lowly expressed genes (genes with 5 or less read counts in at least 9 biological replicates), a total of 12,786 genes were retained for the network analysis.

### Network construction and module identification

**Fig. 1:**
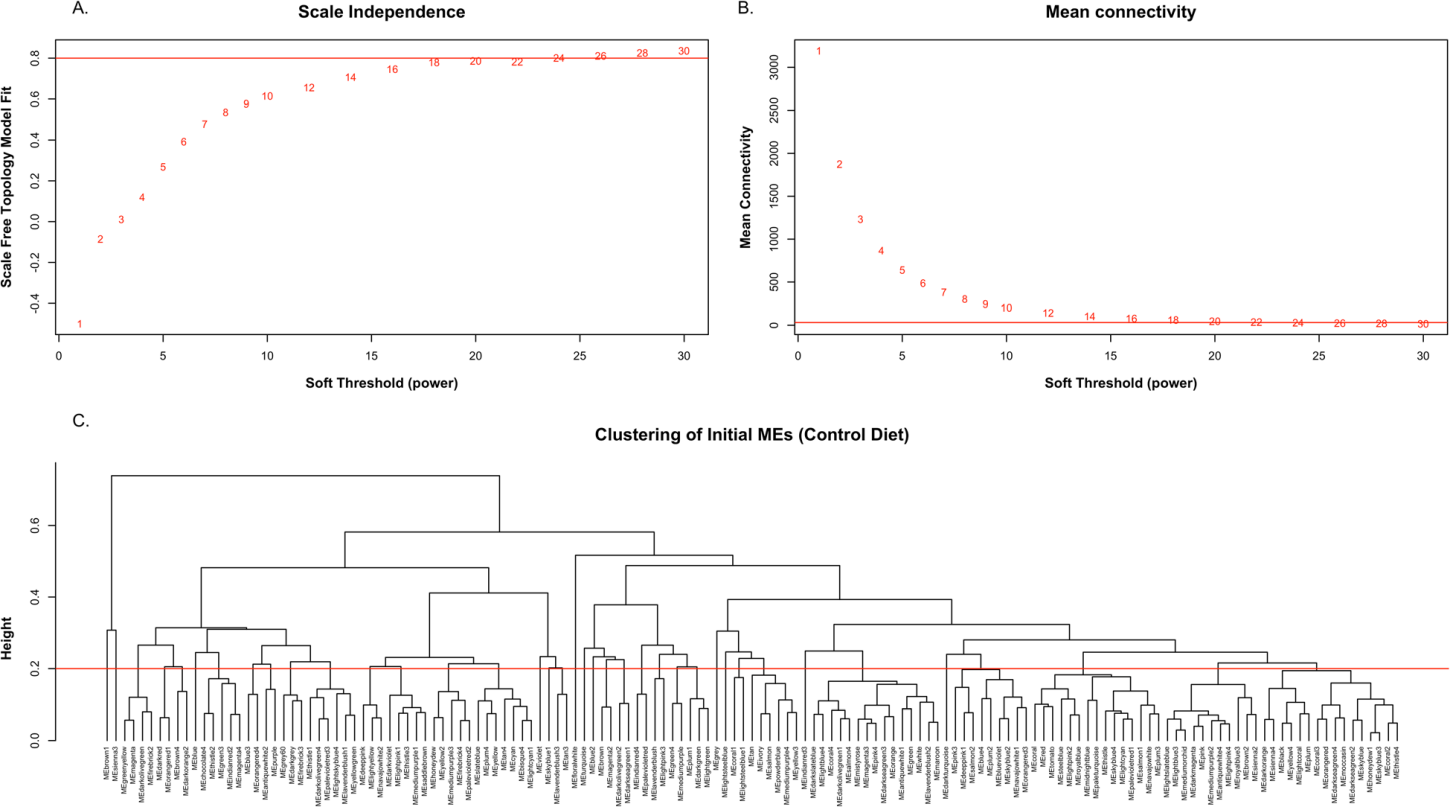
Network topology for various soft-thresholding powers. **a** Scale-free topology fitting index (y-axis) as function of the soft-thresholding power (x-axis). **b** Mean connectivity (y-axis) as function of the soft-thresholding power (x-axis). **c** Tree plot of initial module eigengenes (MEs) in the maternal control diet using soft threshold = 24

**Fig. 2:**
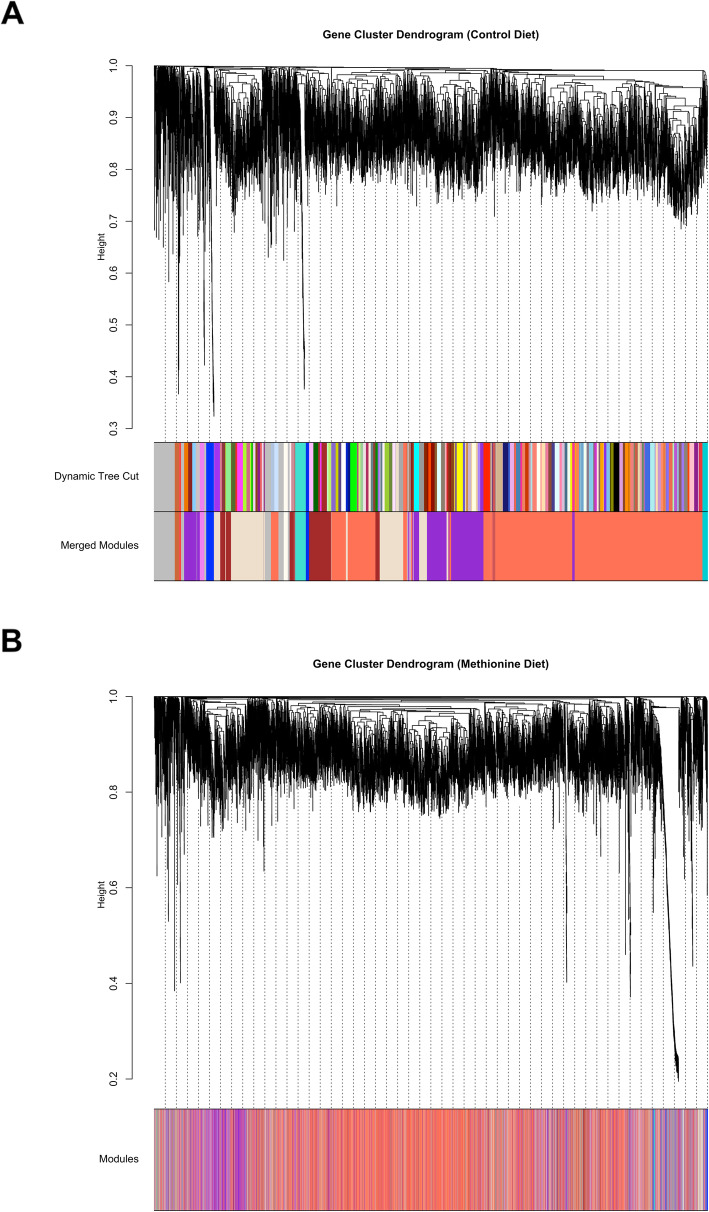
Gene coexpression networks. **a** Gene cluster dendrogram in the maternal control diet. A total of 147 preliminary modules were detected using a dynamic tree cut process, and after merging highly correlated modules (correlation ≥0.8), a total of 14 modules were retained for subsequent analysis. **b** Gene cluster dendrogram in the maternal methionine diet. Changes in the structure of gene coexpression networks between maternal diets were evaluated using a permutation test

We first characterized the muscle transcriptome under normal conditions. As such, the inference of the gene coexpression network and subsequent module identification was performed using only samples derived from the maternal control diet. A total of 7034 genes with high across-sample expression variance were included in this network analysis. A soft-threshold equal to 24 was derived from the high scale-free fitting index (*R*^2^ ≥ 0.8; Fig. 1a), resulting in a mean connectivity equal to 31.45 (Fig. 1b). A total of 147 preliminary modules were detected using a dynamic tree cut process, and after merging highly correlated modules (Pearson’s correlation ≥ 0.8; Fig. 1c), a total of 14 modules, including the background set (grey module) were retained for subsequent analysis (Fig. 2a).

### Module preservation

**Fig. 3:**
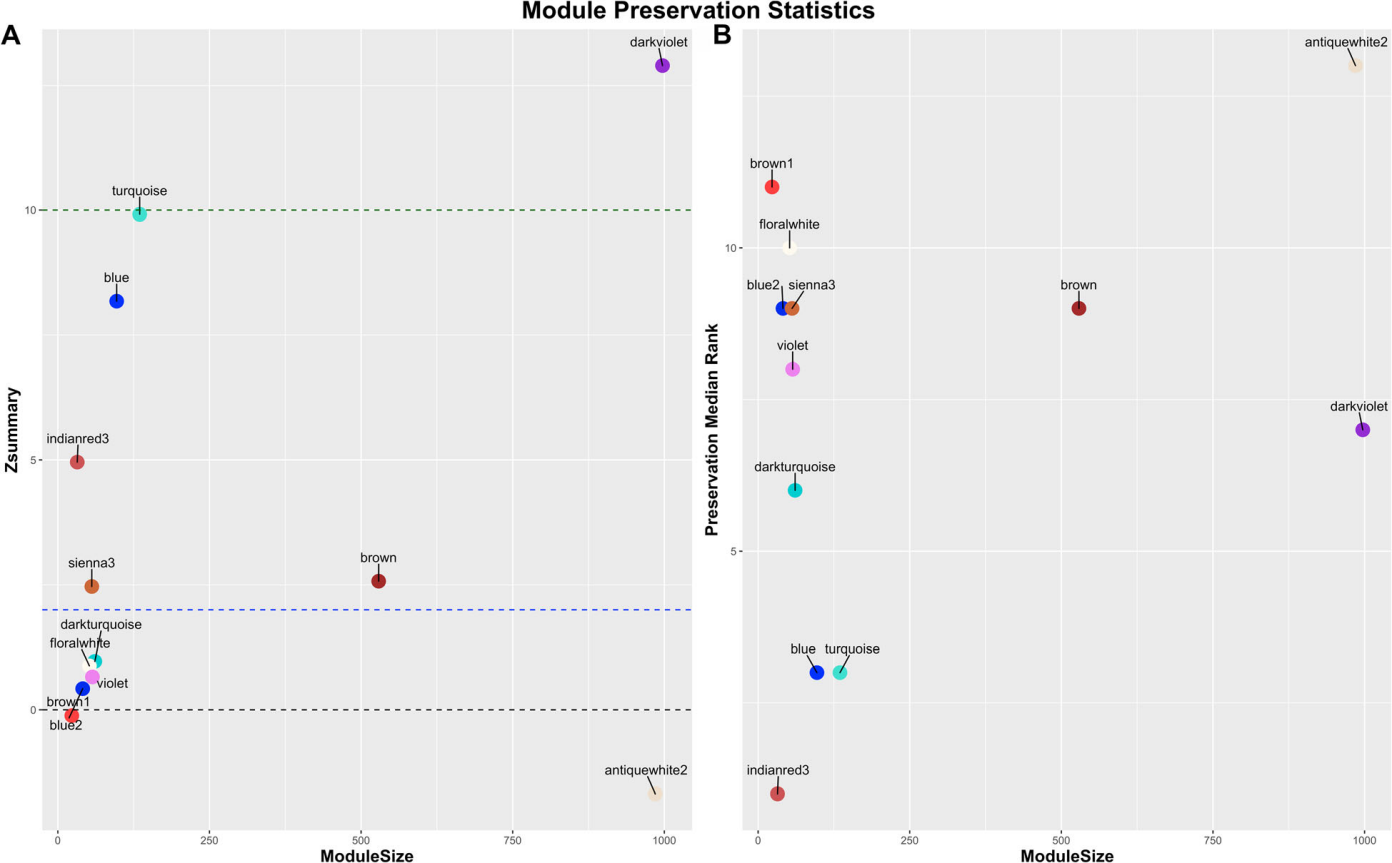
Permutation-based composite preservation statistics. **a** Summary statistics *Z*_*summary*_ (y-axis) as function of module size (number of genes). Non-preserved modules, i.e. subnetworks significantly perturbed by maternal nutrition, were identified using *Z*_*summary*_≤ 2. **b** Summary statistics *medianRank* (y-axis) as function of module size (number of genes). High median rank values suggest strong evidence of perturbation

After we characterized the muscle transcriptome in the control diet, we investigated the impact of the nutritional treatment (maternal methionine supplementation) on gene coexpression patterns. As such, we compared the structure of coexpression networks between control and methionine diets to identify changes in the topology (Fig. 2b). These changes were evaluated using a permutation test with 2000 iterations. Twelve module preservation statistics were calculated for each of the 14 modules previously identified in the control diet (see Additional File 2). A total of six modules were considered as unpreserved (*Z*_*summary*_ ≤ 2), five modules were considered as preserved with weak to moderate evidence of preservation (2 < *Z*_*summary*_ ≤ 10), and finally two modules showed strong evidence of preservation (*Z*_*summary*_ > 10) (Fig. 3). Overall, the six unpreserved modules were considered as gene coexpression modules or subnetworks that were significantly perturbed by the maternal methionine diet.

### Module characterization

**Fig. 4:**
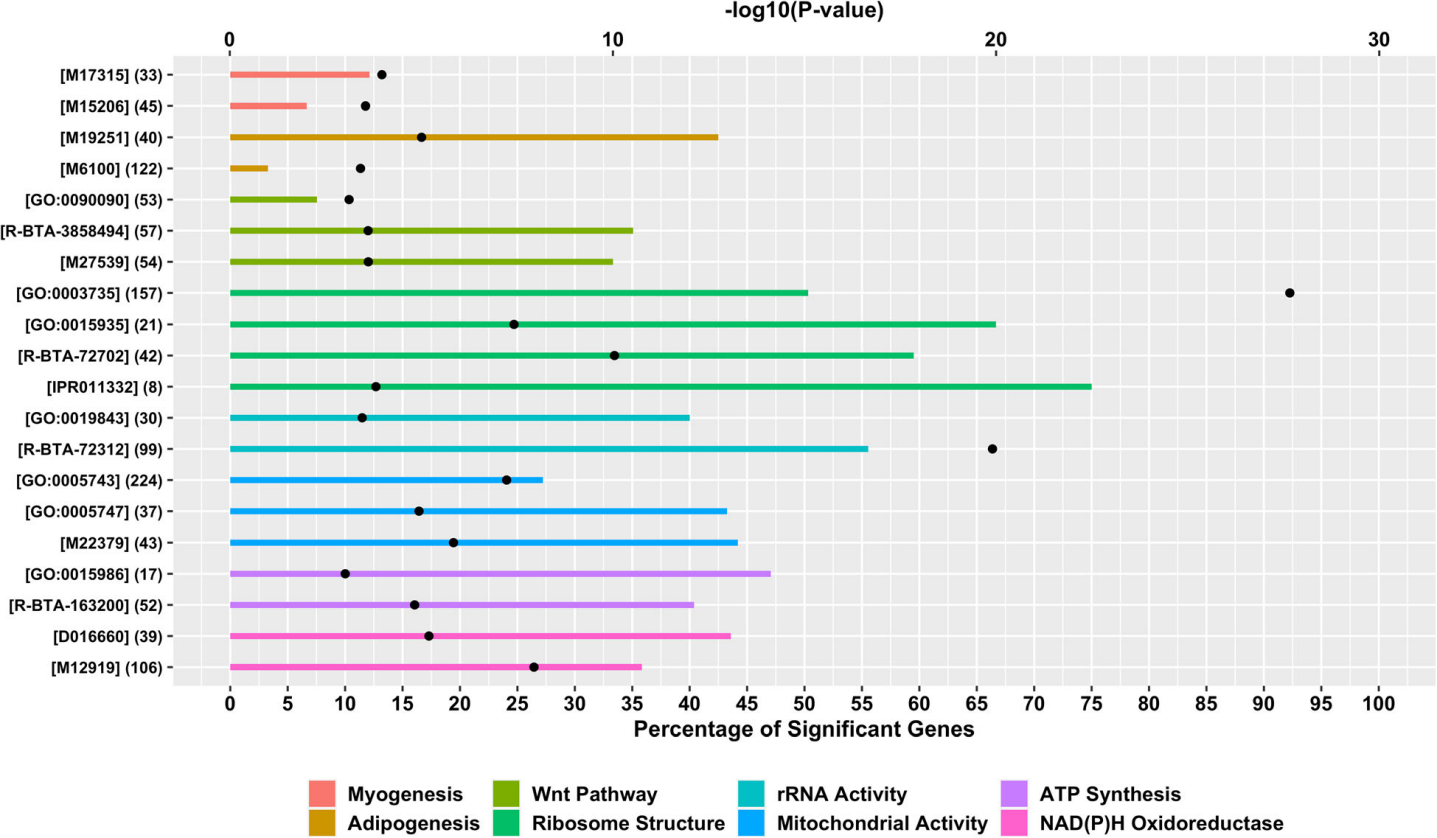
Functional characterization of non-preserved modules. Six gene annotation databases were analyzed: Gene Ontology (GO), Kyoto Encyclopedia of Genes and Genomes (KEGG), Medical Subject Headings (MeSH), InterPro, Reactome and Molecular Signatures Database (MSigDB). The y-axis displays the term ID and the total number of genes in each functional term. The black dots represent the significance of enrichment (Fisher’s exact test, −log_10_ *P*-value, top x-axis) and the bars represent the percentage of significant genes in each functional term (bottom x-axis)

The six unpreserved modules were further investigated to reveal their functional roles and gain insights about the biological processes that were impacted by maternal methionine supplementation. This functional characterization was performed using a Fisher’s exact test, a hypergeometric-based overrepresentation test commonly used to evaluate 2 × 2 contingency tables. Six different biological databases were evaluated, including Gene Ontology (GO), Kyoto Encyclopedia of Genes and Genomes (KEGG), Reactome, InterPro, Medical Subject Headings (MeSH), and Molecular Signatures Database (MSigDB). Figure 4 shows the functional characterization for *antiquewhite2*, the most perturbated module. Interestingly, our analysis revealed that genes in this unpreserved module are closely related to (i) myogenesis, adipogenesis, and fibrogenesis, (ii) ribosome structure, (iii) rRNA binding and processing, (iv) mitochondrial activities, (v) ATP synthesis, and (vi) NAD(P) H oxidoreductases. Moreover, genes in the module *violet* are implicated in the regulation of canonical Wnt signaling pathway, a signal transduction pathway that is involved in different embryonic processes, such as cell fate specification, cell proliferation, and cell migration. Additional File 3 reports the full list of significant functional terms, including term ID, term name, total number of genes in the module, and Fisher’s *P*-value.

### DNA methylation analysis

Whole-genome bisulfite sequencing produced roughly 350 M paired-end reads per sample. The software Bismark was used to map the reads to the ARS-UCD1.2 bovine genome assembly, yielding a 70% mapping rate (see Additional File 1). A total of 5,136,556 cytosines (CpG context) were evaluated (read coverage ≥ 8), and 101,094 were identified as differentially methylated between maternal diets (methylation change ≥ 20%, q-value ≤ 0.10). Based on the ARS-UCD1.2 annotation file, cytosines were classified as (i) within a gene (gene body: exons and introns), (ii) within the regulatory region (5.5 kb upstream the gene), or (iii) located in an intergenic region. As results, we targeted a total of 25,491 genes annotated in the cow genome that had at least one evaluated cytosine (either gene body or regulatory region), and 10,247 of the 25,491 had at least one differentially methylated cytosine. Of interest, a total of 6735 of the 7034 genes used in the network analysis had methylation data. Additional File 4 reports the DNA methylation results, including gene ID and number of cytosines per genomic region. Additional File 5 reports the full list of differentially methylated cytosines and the corresponding genomic regions.

### DNA methylation and module preservation

**Fig. 5:**
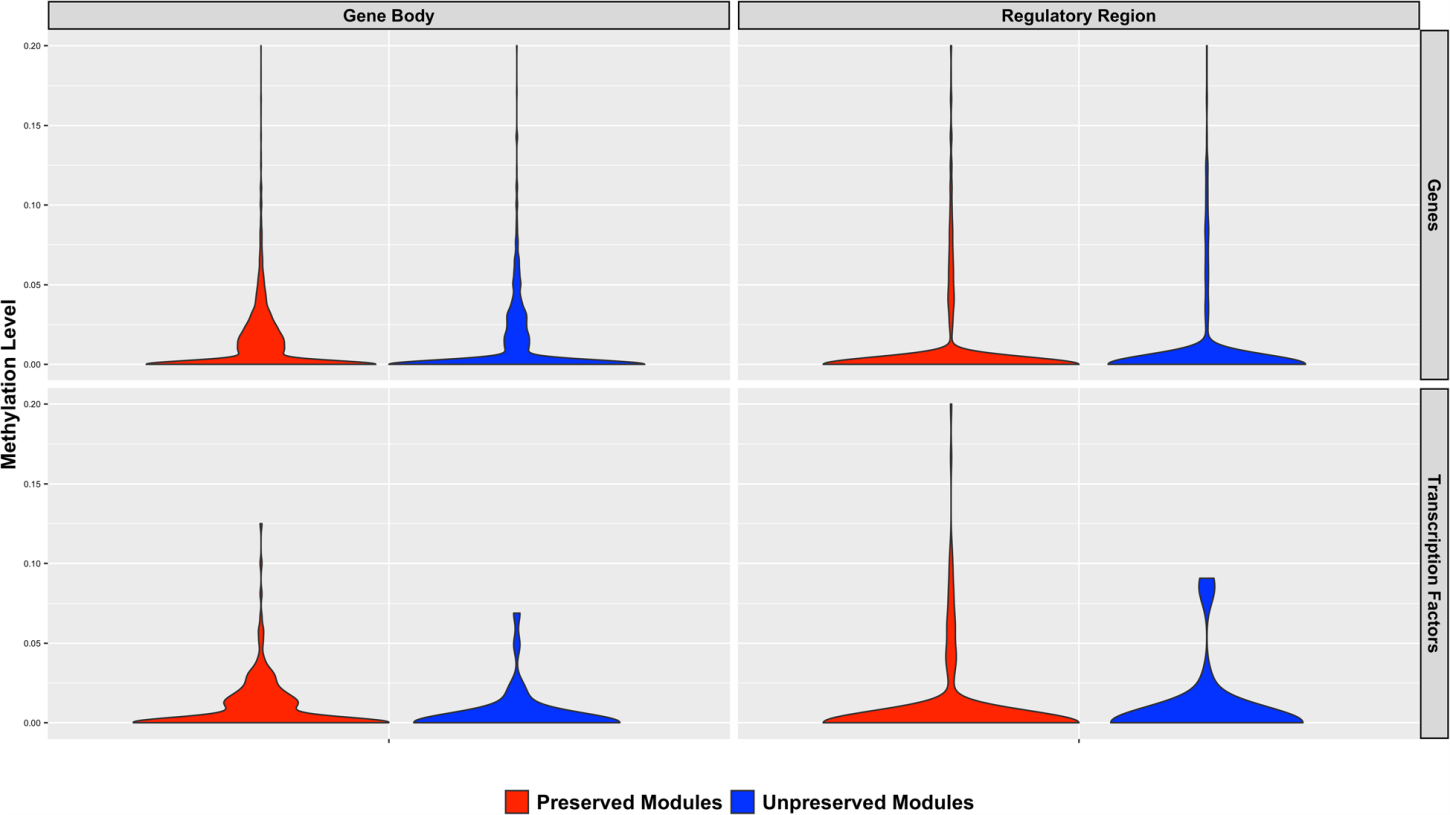
Comparison of methylation levels between preserved and unpreserved modules. Methylation level was calculated as differentially methylated cytosines divided by all the cytosines evaluated. For each gene, including transcription factors, methylation level was evaluated in the regulatory region (transcription start site, promoter and upstream region) and also inside the gene body (exons and introns). The distribution of methylation level in the gene body was different in genes located in unpreserved modules compared to genes in preserved modules (Kolmogorov-Smirnov test, *P*-value ≤ 0.01)

We investigated if there were significant differences in DNA methylation between genes in preserved and unpreserved modules. For each gene, we calculated the methylation level as differentially methylated cytosines divided by all the cytosines evaluated. We calculated the methylation level either for the gene body or the regulatory region. Notably, the distribution of methylation level in the gene body was significantly different in genes located in unpreserved modules (*n* = 1146) compared to genes in preserved modules (*n* = 5589) (Kolmogorov-Smirnov test, *P*-value ≤ 0.01, Fig. 5). We observed the same result if only transcription factors are considered. On the other hand, there was no difference in the distribution of methylation level in the regulatory region between preserved or unpreserved modules, neither for all genes nor for only transcription factors.

### DNA methylation and network properties

**Fig. 6:**
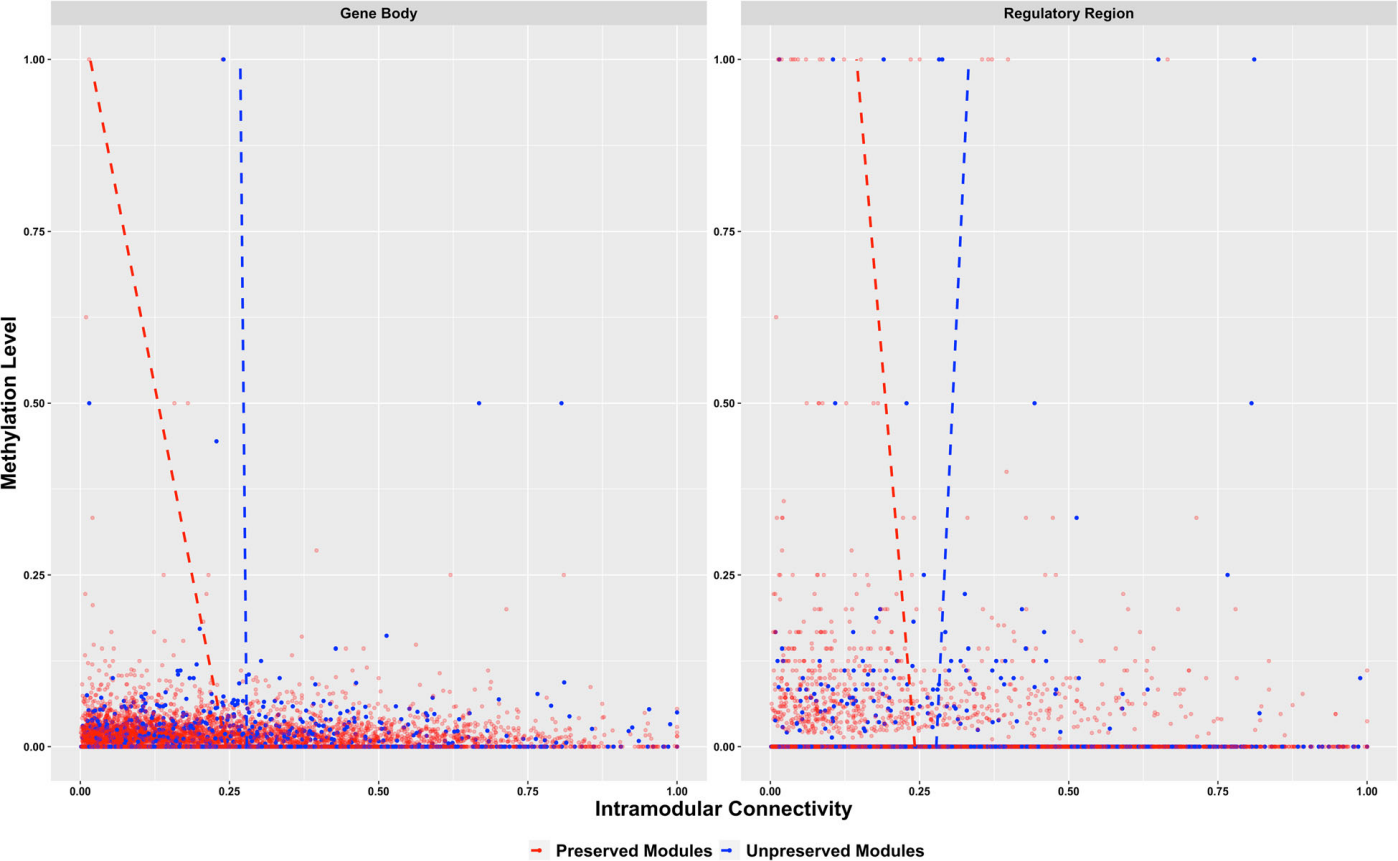
Relationship between methylation level and intramodular connectivity. Methylation level was calculated as differentially methylated cytosines divided by all the cytosines evaluated. For each gene, methylation level was evaluated in the regulatory region (transcription start site, promoter and upstream region) and also inside the gene body (exons and introns). Regressions in preserved modules (red) were significant (*P*-value ≤ 0.05)

We also investigated the relationship between DNA methylation and three different gene network properties, namely differential coexpression score, module membership, and intramodular connectivity. Interestingly, for those genes located in preserved modules, we found a negative relationship between methylation level and intramodular connectivity, considering either the gene body (regression coefficient *β* = −0.23, *P*-value = 0.009, Fig. 6) or the regulatory region (regression coefficient *β* = −0.10, *P*-value = 0.013, Fig. 6). Contrary, there was no relationship (*P*-value > 0.05) between methylation level and intramodular connectivity for genes in unpreserved modules. Moreover, a significant negative relationship was found between methylation level in the regulatory region and module membership (regression coefficient *β* = −0.02, *P*-value = 0.048), but again only for genes in preserved modules. Additional File 6 reports methylation levels and network properties for all the genes evaluated.

## Discussion

Global coexpression network analysis provides a powerful approach to uncover the molecular basis of phenotypic variation. Gene coexpression networks are typically used to infer and annotate gene functions, prioritize candidate regulatory genes, and reveal transcriptional regulatory mechanisms. Lately, there has been greater emphasis on the use of network analysis to elucidate the changes in gene expression patterns in response to changes in experimental conditions or environmental insults. The present study was specially designed to reveal structural changes in gene coexpression networks due to a maternal methionine-rich diet. Maternal nutrition represents a major intrauterine environmental insult that can induce permanent changes in the offspring. Here, we evaluated gene coexpression networks in the muscle of bull beef calves gestated under a control or methionine-rich diet, we functionally characterized the subnetworks altered by maternal methionine supplementation, and we investigated the link between network perturbation and DNA methylation. Our results provide evidence that maternal nutrition can significantly perturb gene coexpression patterns in the offspring, and some of these changes might be mediated by alterations in DNA methylation.

Maternal methionine supplementation significantly disturbed gene coexpression patterns in the offspring’s muscle. In fact, following the methodology proposed by Langfelder and collaborators [5], we identified six modules or subnetworks that significantly changed between experimental conditions. This permutation-based method basically evaluates if the two most important aspects of module topology, namely density and connectivity, are preserved between a reference condition and a test condition. Here, we found that neither the connection strength nor the connectivity pattern of these six subnetworks detected in the control diet were preserved in the methionine-rich diet. There is growing evidence that certain intrauterine insults impact gene coexpression patterns, which in turn may alter fetal development programming. For instance, Deyssenroth et al. [10] reported that alterations in gene coexpression networks in human placenta are associated with abnormal fetal growth and development. Lombardo et al. [11] showed that maternal immune activation via infection during pregnancy disrupts fetal brain gene coexpression networks, and this disruption is associated with an increased risk for autism spectrum disorder. Recently, we reported that exposure to gossypol in utero and during lactation altered the development and gene expression of the testicles, including a significant perturbation of coexpression patterns among spermatogenesis-related genes [12]. Overall, our findings provide further evidence that intrauterine insults, such as diet, not only can change gene expression but also alter coexpression patterns, which in turn suggests alterations in coexpression mechanisms.

The functional characterization revealed that some of the unpreserved modules are directly implicated in myogenesis, adipogenesis, and fibrogenesis. Notably, it is well-documented that maternal nutrition alters fetal skeletal muscle development by interfering with these three important processes [13]. For instance, Zhu and collaborators have shown that nutrient deficiency in ruminants from early to mid-gestation negatively impacts myogenesis, reducing muscle fiber number and also muscle mass [14, 15]. In addition, Tong and collaborators have reported that maternal overnutrition enhances adipogenesis in fetal skeletal muscle [16, 17]. Similarly, Du et al. [13] reported that maternal undernutrition with supplementation of ruminal bypass protein from day 60 to day 180 of gestation significantly affect adipogenesis, changing marbling scores of steer progeny. Moreover, pigs with reduced birth weight due to malnutrition in utero have greater content of collagen in their skeletal muscle [18]. Note that myogenesis, adipogenesis and fibrogenesis are vital aspects of muscle physiology, directly impacting lean muscle mass, marbling and also collagen content, and hence, any disturbances in these processes may have long-term consequences, impacting muscle growth and meat quality.

We also found unpreserved subnetworks related to regulation of canonical Wnt/β-catenin pathway. By acting through autocrine and/or paracrine mechanisms, the Wnt family of secreted glycoproteins affects different aspects of cell physiology, such as cell proliferation, cell differentiation or maintenance of precursor cells [19, 20]. Interestingly, in skeletal muscle, β-catenin regulates the expression of two transcription factor, namely *PAX3* and *GLI*, which are essential for skeletal myogenesis [21, 22]. Indeed, some studies have shown that blocking the β-catenin pathway reduces the total number of myocytes [23, 24]. Shang and colleagues showed that the upregulation of Wnt signaling promotes myogenesis, whereas downregulation of this pathway promotes adipogenesis [25]. Moreover, we identified terms related to basic cell structures/functions, including ribosome structure, rRNA binding and processing, mitochondrial activities, ATP synthesis and NAD(P) H oxidoreductases. Notably, previous studies have shown that these cell activities can be affected by maternal nutrient restrictions. For instance, Peñagaricano et al. [26] reported that maternal diets significantly impact functional terms closely related to ribosome in fetal muscle. Moreover, Mayeur et al. [27] found that maternal undernutrition induces placental mitochondrial abnormalities and reduced ATP level in mice offspring. Similarly, Zhu et al. [14] found that maternal nutrient restriction can induce downregulation of key enzymes involved in mitochondrial function in offspring’s muscle.

The exact mechanisms by which maternal diet can affect gene coexpression patterns in the offspring are not yet known. Here, we examined the hypothesis that changes in DNA methylation cause changes in the topology of gene networks. Nearly 2% of all the evaluated cytosines in a CpG context were found to be differentially methylated between maternal diets. Notably, we found significant differences in the level of gene body methylation between genes in preserved modules versus genes in unpreserved modules. The same trend was observed when only transcription factors were considered. Despite the function of DNA methylation in regulatory regions is well-known, the role of DNA methylation within the gene is not yet well understood. Some studies have suggested that DNA methylation in gene body might be involved in the regulation of alternative splicing [28, 29]. Of special interest, Saha et al. [30] reported that the regulation of alternative splicing is coordinated across functionally related genes. Therefore, changes in DNA methylation might cause changes in isoform expression, which in turn can alter gene coexpression patterns. Moreover, changes in DNA methylation also altered subnetwork properties. Indeed, in preserved modules, there was a clear negative relationship between methylation level and intramodular connectivity, i.e., more methylation, less connectivity, that is, less gene activity. Notably, this functional relationship completely disappeared in unpreserved modules. Overall, our findings suggest that maternal methionine supplementation may induce changes in the offspring epigenome, such as changes in DNA methylation, which in turn alter coexpression patterns and gene network properties.

## Conclusions

Our study has shown that maternal nutrition levels during preconception and early pregnancy can significantly impact gene coexpression patterns in the offspring. Some of the perturbed gene functions are directly implicated in the development of skeletal muscle, such as myogenesis, adipogenesis, and Wnt/β-catenin pathway. Notably, some of the changes in gene coexpression patterns are associated with changes in DNA methylation. To the best of our knowledge, this is the first study that investigates the link between maternal nutrition, DNA methylation and gene coexpression networks. Our findings suggest that maternal nutrition perturbs gene coexpression patterns, and these alterations are in part mediated by changes in the epigenome.

## Methods

### Ethics statement

All the animal procedures used in this study were approved by the Institutional Animal Care and Use Committee (IACUC #2014408583) of the University of Florida. All experiments were performed in accordance with relevant guidelines and regulations.

### Animals and experimental design

Brangus-Angus crossbred beef cows from the University of Florida Range Cattle Research and Education Center (Ona, Florida, US) were assigned to one of two nutritional treatments from days − 30 to + 90 relative to the beginning of the breeding season. These treatments consisted of a control diet based on limpograss hay (*Hemarthria altissima*) supplemented with molasses and urea (22% crude protein, 1.7 kg per head per day) and a methionine-rich diet equal to the control diet but supplemented with 10 g per head per day of MetaSmart Liquid (Adisseo, Alpharetta, GA) providing 3.7 g per head per day of rumen-protected methionine. Longissimus dorsi muscle samples were collected from 20 bull calves, 10 per maternal diet, at 1 month of age. After sampling, the bull calves were released. Maternal diets did not affect birth or weaning weight, but altered post weaning calf growth performance, calves derived from the methionine-rich diet had greater average daily gain (0.985 kg vs 0.810, *P*-value = 0.043) and also greater gain-to-feed ratio (0.191 vs 0.159, *P*-value = 0.025) post weaning.

### RNA extraction, library preparation and sequencing

Total RNA was extracted using Qiagen RNeasy Mini kit. RNA yield and quality were evaluated using the Agilent 2100 Bioanalyzer (Agilent Technologies, Inc.). RNA-sequencing libraries were prepared from 50 ng RNA samples using a poly(A) capture method and then sequenced using Illumina’s HiSeq 3000 at the University of Florida. A total of 19 muscle samples from 19 bull calves derived from 9 control and 10 methionine-rich maternal diets were successfully processed and sequenced, and hence used for subsequent RNA-sequencing analyses. RNA-sequencing data can be accessed by NCBI GEO with the accession number GSE116974.

### RNA-seq quality control and mapping

The quality of the sequencing reads was evaluated using the software FastQC (v0.11.7, Babraham Bioinformatics, UK). Adaptor removal and trimming were conducted with Trim Galore (version 0.4.4, Babraham Bioinformatics, UK) using the following parameters: --paired, −-clip_R1 10, −-clip_R2 10, −-three_prime_clip_R1 10, −-three_prime_clip_R2 10, and --length 20. The resulting paired-end sequencing reads were mapped to the latest bovine reference genome (ARS-UCD1.2) using the software Hisat2 (v2.1.0) [31].

### Read counting, processing and normalization

Number of reads that mapped to each annotated gene in the bovine GTF file was obtained using the python script *htseq-count* (v0.6.1p1) using the option *intersection-nonempty* [32]. Both highly abundant genes (*n* = 25 genes, such as myosins, tropomyosins, and troponins) and lowly expressed genes (read counts ≤ 5 in at least 9 biological replicates) were removed from the raw expression data and not included in subsequent analyses. After data processing, read counts were normalized using the trimmed mean of M-values (TMM) normalization method available in the *R* package edgeR (v3.14) [33].

### Gene coexpression network construction

Genes with high expression variance across samples (top 50%) were used for network modeling. The *R* package WGCNA (v1.69) was used for network construction [34, 35]. First, an unsigned adjacency matrix was constructed based on pairwise Pearson correlation coefficients using the function *adjacency*. This adjacency matrix was then remodeled as Topological Overlap Matrix (TOM) using the function *TOMsimilarity*. The TOM-based dissimilarity matrix, simply defined as *dissTOM* = (1 – *TOM*), was used as pairwise distance matrix for hierarchical clustering. Note that genes in the same cluster share strong interconnections, and they might define modules or subnetworks. Module detection was performed by cutting the branches of the clustering dendrogram using the function *cutreeDynamic*. Genes that could not be assigned to any module were considered as background genes (grey module) and were not included in subsequent module preservation analyses. For each module, the variable module *eigengene* (ME) was calculated as the first principal component of the module expression data. Modules detected in the dynamic cutting process were merged if their eigengene values were highly correlated (Pearson correlation ≥ 0.8). The module membership was calculated using the function *signedKME*, where the expression profile of each gene was correlated with the module eigengene in order to quantify how connected a gene was to a given module.

### Module preservation

The function *modulePreservation* implemented in the *R* package WGCNA was used to evaluate the preservation of each module (subnetwork) across conditions, i.e. between maternal control and maternal methionine-rich diets. A total of 12 different module preservation statistics were investigated using a permutation test with 2000 resamples. These 12 different preservation statistics were combined in two composite preservation significance scores, namely *Z*_*density*_ and *Z*_*connectivity*_. Finally, *Z*_*summary*_ was computed as the mean of *Z*_*density*_ and *Z*_*connectivity*_, representing the general preservation status of a given module across the two experimental conditions [5]. In addition, an alternative module-size-independent statistic *medianRank* was also calculated to assess module preservation. This rank-based statistic is defined as the mean of observed median ranks for individual connectivity and density preservation statistics in each module, and thus modules with low median rank values are considered as preserved between conditions/treatments. Finally, following Langfelder and colleagues, an unpreserved module was defined as a module with *Z*_*summary*_≤ 2 and *medianRank* greater than half of the total number of the modules detected [5].

### Module characterization

The functional characterization of the unpreserved modules was performed using a Fisher’s exact test, a test of proportions based on the cumulative hypergeometric distribution. Genes within each unpreserved module were scrutinized using six different databases, including *Gene Ontology* (GO) [36], *KEGG* [37], *Interpro* [38], *Reactome* [39], *Medical Subject Headings* (MeSH) [40] and *Molecular Signatures Database* (MSigDB) [41]. This over-representation analysis, i.e., evaluate whether a given biological pathway, molecular function or functional term is enriched or overrepresented with genes in the unpreserved module, was performed using the *R* package *EnrichKit* (https://github.com/liulihe954/EnrichKit).

### DNA extraction, library preparation and sequencing

Total DNA was extracted from muscle samples for whole-genome bisulfite sequencing analysis. Extraction, library construction, bisulfite treatment and sequencing were performed by Novogene Bioinformatics Technology Co., Ltd. (Beijing, China). Libraries were sequenced with Illumina’s HiSeq 3000 using 150-bp paired-end reads. A total of 16 muscle samples from 16 bull calves derived from 7 control and 9 methionine-rich maternal diets were successfully processed and sequenced, and hence used for subsequent bisulfite-sequencing analyses. Whole-genome bisulfite sequencing data can be accessed by NCBI GEO with the accession number GSE117194.

### Bisulfite-seq quality control and mapping

The quality of the sequencing reads was evaluated using the software FastQC (v0.11.7, Babraham Bioinformatics, UK). Adaptor removal and trimming was performed when needed using the software Trim Galore (v0.4.4, Babraham Bioinformatics, UK). After quality control and processing, the resulting paired-end sequencing reads were aligned to ARS-UCD1.2 bovine reference genome using the software Bismark (v0.17.0, Babraham Bioinformatics, UK) [42]. The tool *deduplicate_bismark* was used to remove duplicate read alignments. Methylation calls were performed using Bismark *methylation extractor* (v0.17.0, Babraham Bioinformatics) using the following parameters: --paired-end, −-comprehensive, −-bedGraph, and --cytosine_report [42].

### Differentially methylated cytosines and genes

Differential methylation between maternal diets was analyzed using a logistic regression implemented in the *R* package Methylkit (v1.0.0) [43]. Only cytosines with read coverage equal or greater than 8 in a CpG context were evaluated. Differentially methylated cytosines were defined as those having methylation percentage changes between treatments greater than 20% and q-values ≤0.10. The software *Rgmatch* was used to match the cytosines to different gene features, such as transcription start site, exons, introns, and upstream regions [44]. In addition, we defined methylation level as the ratio of differentially methylated cytosines to all the cytosines evaluated in a given region. For each gene, we calculated the methylation level for the regulatory region (transcription start site, promoter and upstream region) and also inside the gene body (exons and introns).

## Supplementary information

**Additional file 1.** Mapping Stats.xlsx: Summary of sequencing read alignments to the reference genome for both RNA-Sequencing and Whole-Genome Bisulfite-Sequencing.

**Additional file 2.** Module Preservation Stats.xlsx: Summary of composite and individual module preservation statistics.

**Additional file 3.** Overrepresentation Summary.xlsx: List of significant functional terms.

**Additional file 4.** Gene CpG Count by Region.xlsx: Count of cytosines (CpG context) evaluated in each of the six genomic regions per gene.

**Additional file 5.** Associations of Differentially Methylated CpGs.xlsx: List of differentially methylated cytosines.

**Additional file 6.** Gene Methylation level and Network Properties Measurements.xlsx: Summary of statistics at gene level including differential coexpression score, connectivity, module eigengenes, count of differentially methylated cytosines and methylation levels.

## Data Availability

Bisulfite-Seq (GSE117194) and RNA-Seq (GSE116974) data are available on NCBI GEO with accession number (SuperSeries) GSE117195. The ARS-UCD1.2 bovine reference genome is available on Ensembl: ftp://ftp.ensembl.org/pub/release-101/fasta/bos_taurus/dna/.

## Abbreviations

GO: Gene Ontology
KEGG: Kyoto Encyclopedia of Genes and Genomes
ME: Module eigengene
MeSH: Medical Subject Headings (MeSH)
MSigDB: Molecular Signatures Database
RNA-seq: RNA-sequencing
TMM: Trimmed mean of M-values
TOM: Topological Overlap Matrix
